# Feline XLF accumulates at DNA damage sites in a Ku‐dependent manner

**DOI:** 10.1002/2211-5463.12589

**Published:** 2019-05-21

**Authors:** Manabu Koike, Yasutomo Yutoku, Aki Koike

**Affiliations:** ^1^ National Institute of Radiological Sciences National Institutes for Quantum and Radiological Science and Technology Chiba Japan

**Keywords:** companion animal, DNA double‐strand break, feline, Ku80, non‐homologous DNA‐end joining, cat

## Abstract

Resistance to radiotherapy and chemotherapy is a common problem in the treatment of cancer in humans and companion animals, including cats. There is thus an urgent need to develop new treatments. Molecularly targeted therapies hold the promise of high specificity and significant cancer‐killing effects. Accumulating evidence shows that DNA double‐strand break (DSB) repair proteins, which function in Ku‐dependent non‐homologous DNA‐end joining (NHEJ), are potential target molecules for next‐generation cancer therapies. Although cancer radioresistance in cats has been previously described, there are no reports on feline Ku‐dependent NHEJ. Here, we cloned and sequenced feline *XLF* cDNA and characterized X‐ray repair cross‐complementing protein 4‐like factor (XLF), which is one of the core NHEJ proteins. We demonstrated that feline XLF localizes to the nuclei of feline cells and that feline XLF immediately accumulates at laser‐induced DSB sites in a Ku‐dependent manner. Amino acid sequence alignment analysis showed that feline XLF has only 80.9% identity with human XLF protein, while the predicted nuclear localization signal and putative 14‐3‐3‐binding motif are perfectly conserved among human, cat, dog, chimpanzee, and mouse. These findings are consistent with the hypothesis that regulation of subcellular localization is important for the function of XLF. Furthermore, these findings may be useful in clarifying the mechanisms underlying feline Ku‐dependent DSB repair and feline cell radioresistance, and possibly facilitate the development of new molecularly targeted therapies that target common proteins in human and feline cancers.

**Abbreviations**
A‐EJ: alternative end joining
ATM: ataxia telangiectasia mutated
β‐TRCP: β‐transducin repeat containing protein
CKI: casein kinase I
DIC: differential interference contrast
DNA‐PKcs: DNA‐dependent protein kinase, catalytic subunit
DSB: DNA double‐strand break
HR: homologous recombination
HRP: horseradish peroxidase
IR: ionizing radiation
NHEJ: non‐homologous DNA‐end joining
NLS: nuclear localization signal
PAXX: PAralog of XRCC4 and XLF
PD‐1: anti‐programmed cell death protein 1
PD‐L1: programmed cell death 1 ligand 1
PTM: post‐translational modification
XLF: X‐ray repair cross‐complementing protein 4‐like factor
XRCC4: X‐ray repair cross‐complementing protein 4

Companion animals are of tremendous importance in the lives of many people. Unfortunately, about 6 million dogs and a similar number of cats are diagnosed with cancer in the USA each year 1. Nickoloff *et al*. 2 described that companion animal studies, under the umbrella of comparative oncology, have played key roles in the development of clinical radiotherapy throughout its more than 100‐year history. They also mentioned that canine cancer models present many translational research opportunities to exploit fundamental knowledge about DNA repair to improve radiotherapy 2. Radiation is becoming widely available to treat tumors in companion animals such as cats and dogs 3. However, the effect of radiation therapy in cats seems to be very different from in dogs in terms of tumor responses as well as normal tissue toxicity. Radioresistance of cats has been observed in animal radiotherapy at veterinary hospitals 3. In addition, at the cellular level, feline normal fibroblasts were more radioresistant than human fibroblasts 4. The feline cells displayed a decreased residual amount of DNA double‐strand breaks (DSBs) after potential lethal damage repair, suggesting that DNA damage induced by X‐irradiation is more effectively repaired in feline cells. Thus, chemo‐radiotherapy is expected to be one of the most effective treatments, not only for companion animals, but also for humans, if the next‐generation radiosensitizers specifically targeting cancer cells in cats can be developed.

Molecularly targeted therapies of cancer promisingly have a high selectivity and significant cancer‐killing effects. The DSBs are the most dangerous DNA damage 2. There are three pathways, namely non‐homologous DNA‐end joining (NHEJ), homologous recombination (HR) and alternative end joining (A‐EJ), for repairing DSBs in human and other mammalian cancer cells. An accessory NHEJ factor, DNA‐dependent protein kinase, catalytic subunit (DNA‐PKcs), has been reported to be upregulated in human tumors and radiation‐resistant cell lines, suggesting that this protein has a role in tumor growth and survival 5, 6, 7, 8. Thus, the NHEJ factors including DNA‐PKcs are potential targets of drug discovery for the next‐generation cancer therapies 5, 6, 7, 8. Recently, Gemenetzidis *et al*. 9 suggested that oral cancer stem cells display resistance to ionizing radiation (IR), and this correlates with elevated levels of X‐ray repair cross‐complementing protein 4 (XRCC4)‐like factor (XLF), which is the other core NHEJ factor. Hence, XLF might be a pharmacological target in cancer therapy. However, as yet there are no drugs targeting XLF.

It is important to uncover the regulatory mechanisms of DNA repair in human and companion animal cells to develop novel molecularly targeted therapies 10, 11. Post‐translational modifications (PTMs) and protein–protein interactions might trigger and control DNA repair processes and DNA damage response signals to repair DSBs efficiently. Recently, we cloned and characterized four cDNAs of the canine core NHEJ repair genes 12, 13, 14, 15. However, there are no reports about mechanism of DNA repair in feline cells. Furthermore, the sequence, localization, and regulatory mechanisms of each feline core NHEJ factor, e.g. XLF or Ku70, have not been published. In this study, we first cloned feline *XLF* cDNA and examined its expression, localization, and recruitment to DSB sites of feline XLF proteins. In addition, we carried out comparative analysis to uncover the regulatory mechanisms which govern XLF's functions.

## Materials and methods

### Cloning of feline XLF

Cloning of feline XLF cDNA was performed as previously described 12, 13 with the following modifications. Oligonucleotide primers used to amplify feline *XLF* cDNA from a male cat cDNA library (Zyagen, San Diego, CA, USA) were designed based on the predicted XLF genomic sequence of female cat, belonging to the species *Felis catus* (XM_011285546.1). PCR amplification with sense (feline XLF F1: 5′‐GAATTCTATGGAGGAACTGGAGCAAGGTCTG‐3′) and antisense (feline XLF R: 5′‐GGATCCTTAACTGAAGAGCCCCCTTAGCTTC‐3′) primers was performed in a TaKaRa PCR Thermal Cycler Dice (Takara Bio Inc., Otsu, Japan) using LA Taq polymerase (Takara Bio Inc.). Pre‐denaturation was carried out for 5 min at 94 °C. This was followed by 35 cycles of PCR amplification. Each cycle consisted of a denaturation step at 94 °C for 0.5 min, annealing at 56 °C for 0.5 min and extension at 72 °C for 0.5 min, followed by a final extension step (4 min). PCR products were subcloned into the pCR4‐TOPO vector (Thermo Fisher Scientific, Waltham, MA, USA) (pCR4‐feline *XLF* plasmid), and the nucleotide sequences were determined by sequencing using primers, T3 and T7. XLF cDNA from pCR4‐feline *XLF* plasmid was subcloned into the EcoRI and BamHI sites of pEYFP‐C1 (pEYFP‐feline *XLF*), and the inserts were validated by sequencing. Other PCR primers used in this study were as follows: feline XLF F11: 5′‐CTCTAGGCCTTTCGGTTTGC‐3′, feline XLF R11: 5′‐GCGAAGCAGATCATCCAAAT‐3′, feline XLF F2: 5′‐CCCCACAAGGAACTGAAAACCAAC‐3′ and feline XLF R2: 5′‐CCTTTTAGGCTGACATTAGGGCAC‐3′. These PCR primers were used to validate that the above synthetic primer sequence (feline XLF F1) is the true sequence. We confirmed that the sequence around ATG is based on the cognate sequence.

### Cell lines, cultures and transfections

A human colon cancer cell line (HCT116, Riken Cell Bank, Tsukuba, Japan), a Chinese hamster ovary cell line (CHO‐K1, Riken Cell Bank), and a Ku80‐deficient CHO‐K1 mutant cell line (xrs‐6) were cultured as described in previous studies 15, 16, 17. A Crandell‐Reese feline kidney (CRFK) cell line (HSRRB, Osaka, Japan) and a XLF‐deficient cell line HCT116 (XLF^−/−^) (Horizon, Cambridge, UK) were cultured in Dulbecco's modified Eagle's medium with 10% fetal bovine serum. The pEYFP‐feline *XLF* or pEYFP‐C1 was transiently transfected into cells using Lipofectamine 3000 (Thermo Fisher Scientific). Post‐transfection, cells were cultured for 2 days and then examined under an FV300 confocal laser‐scanning microscope (Olympus, Tokyo, Japan), as previously described 18, 19.

### X‐irradiation

X‐irradiation was carried out as described previously 13. Cells were exposed to X‐rays at 10 Gy at a dose rate of 0.72 Gy·min^−1^ using the Pantak HF320S X‐ray system (Shimadzu, Kyoto, Japan) operating at 200 kV, 20 mA with a filter of 0.5 mm aluminum and 0.5 mm copper.

### Western blot analysis

The extraction of total cell proteins and western blot analysis were carried out as described previously 14, 15, 16, 17 with the following modifications. The molecular mass marker used was 3‐Color Prestained XL‐Ladder (APRO science, Tokushima, Japan). The membranes were blocked in Blocking One (Nacalai Tesque, Kyoto, Japan) or ECL Prime Blocking reagent (GE Healthcare Bio‐Sciences Corp., Piscataway, NJ, USA) for 30 min at room temperature. The following antibodies were used: rabbit anti‐Ku80 polyclonal antibody (AHP317; Serotec, Oxford, UK), mouse anti‐Ku80 monoclonal antibody (B‐4, Santa Cruz Biotechnology, Santa Cruz, Dallas, TX, USA), rabbit anti‐Ku80 monoclonal antibody (H‐300, Santa Cruz Biotechnology), rabbit anti‐Ku70 polyclonal antibody (H‐308, Santa Cruz Biotechnology), mouse anti‐Ku70 monoclonal antibody (A‐9, Santa Cruz Biotechnology), goat anti‐XLF polyclonal antibody (SAB2501119, Sigma‐Aldrich, St Louis, MO, USA), rabbit anti‐XLF polyclonal antibody (A300‐730A) (Bethyl Laboratories, Montgomery, TX, USA), mouse anti‐γH2AX monoclonal antibody (JBW301, Upstate Biotechnology Inc., Charlottesville, VA, USA), rabbit anti‐H2AX polyclonal antibody (A300‐083A, Bethyl Laboratories), rabbit anti‐GFP polyclonal antibody (FL, Santa Cruz Biotechnology), goat anti‐GFP polyclonal antibody (AB0020, SICGEN, Carcavelos, Portugal), and mouse anti‐β‐actin monoclonal antibody (Sigma‐Aldrich). The following secondary antibodies were used: anti‐mouse IgG, horseradish peroxidase (HRP)‐linked whole Ab sheep (NA931, GE Healthcare Bio‐Sci. Corp.), anti‐rabbit IgG, HRP‐linked whole Ab donkey (NA934, GE Healthcare Bio‐Sci. Corp.), and donkey anti‐sheep/goat IgG antibody, HRP conjugate (AB324P, Millipore, Billerica, MA, USA). The binding to each protein was detected using a Select western blotting detection system (GE Healthcare Bio‐Sci. Corp.) in accordance with the manufacturer's instructions, and visualized using the ChemiDoc XRS system (Bio‐Rad, Hercules, CA, USA).

### DNA damage induction using micro‐laser and cell imaging

Local DNA damage induction using microlaser and subsequent cell imaging was carried out as described previously 15, 19. Briefly, local DSBs were induced using a 405 nm laser. Images of living or fixed cells expressing EYFP–feline XLF or EYFP alone were obtained using an FV300 confocal laser‐scanning microscope system (Olympus). Immunocytochemistry was carried out using rabbit anti‐Ku80 polyclonal antibody (AHP317), rabbit anti‐Ku70 polyclonal antibody (H‐308), a mouse anti‐γH2AX monoclonal antibody (JBW301), and Alexa Fluor 568‐conjugated secondary antibody (Thermo Fisher Scientific), as previously described 15, 19.

## Results

### Phosphorylation of H2AX after X‐irradiation and expression of core NHEJ factors in feline cells

**Figure 1:**
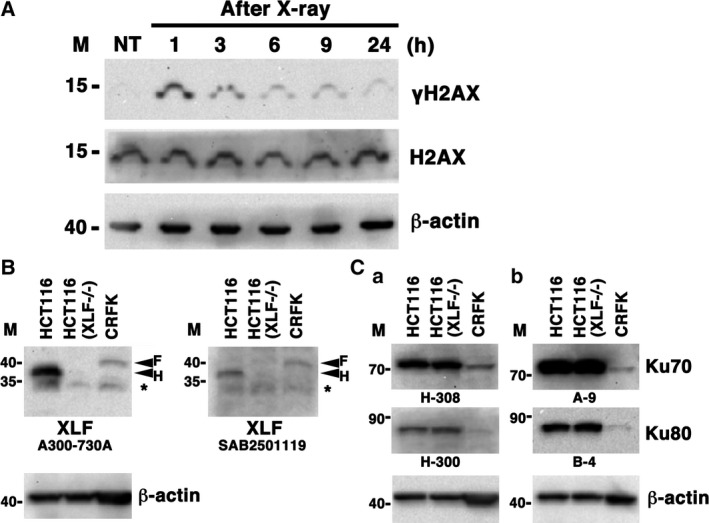
Phosphorylation of H2AX in feline cells after X‐irradiation. (A) The feline CRFK cells were either non‐irradiated (NT) or irradiated with 10 Gy X‐rays. Each of the total protein samples from the cells was prepared 1, 3, 6, 9, or 24 h after X‐irradiation. γH2AX protein level was determined by western blot analysis using a specific antibody against γH2AX. β‐Actin and H2AX were used as the loading control. (B,C) Expression of core NHEJ factors in feline and human cells. Total cell proteins from the feline cell line (CRFK, 50 μg/lane) and human cell line [HCT116 and HCT116(XLF ^−/−^), 20 μg/lane (B), 10 μg/lane (C)] were analyzed by western blotting using two anti‐XLF antibodies (A300‐730A and SAB2501119) (B), two anti‐Ku80 antibodies (B‐4 and H‐300) (C), two anti‐Ku70 antibodies (H‐308 and A‐9) (C), or anti‐β‐actin antibody (B,C). Sequential detections of XLF and β‐actin were performed by reprobing on the same blot (B). A blot was cut into two pieces, and sequential detection of Ku70 and Ku80 was performed on the same piece, and β‐actin was detected on another piece (Ca,b). Arrowheads show XLF of cat (F) or human (H). *Non‐specific band. M, molecular mass marker (kDa).

Histone H2AX is rapidly phosphorylated at serine 139 (γH2AX) in response to DSBs, and the reduction of γH2AX reflects DSB repair 20. To test whether the DSB repair pathways are intact in feline CRFK cells, we examined X‐irradiation‐induced H2AX phosphorylation and γH2AX reduction in extracts from CRFK cells by western blot analysis using the anti‐γH2AX antibody. As shown in Fig. 1A, a high level of γH2AX was detected in extracts from CRFK cells at 1 h post‐irradiation, and γH2AX reduction was detected from 1 to 6 h after X‐irradiation. Mammalian cells have three DSB repair pathways, i.e. NHEJ, HR, and A‐EJ, and NHEJ, but not HR and A‐EJ, contributes to the fast repair within hours after irradiation to X‐ray‐induced DSBs 2, 6. We speculate that the fast DSB repair pathway, i.e. NHEJ, might be intact in CRFK cells.

Next, we examined the expression of core NHEJ factors, i.e. Ku70, Ku80, and XLF in feline (CRFK) and human [HCT116 and HCT116 (XLF^−/−^)] cell lines. As shown in Fig. 1B,C, using each of two specific antibodies, the expression of feline XLF, Ku70 and Ku80 was detected in CRFK cells. Interestingly, the size of the feline XLF protein was greatly distinct from that of human. We speculate that the PTMs on feline XLF are different from those of human, although further studies need to clarify this.

### Cloning and sequence analysis of feline XLF

**Figure 2:**
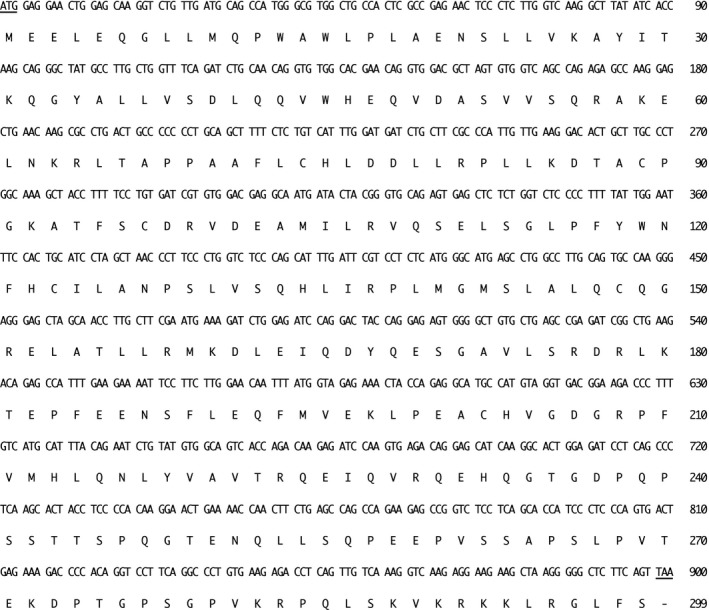
Nucleotide and deduced amino acid sequences of feline *XLF* cDNA (*Felis catus*, GenBank accession number: LC309246). The coding sequence of feline *XLF* is composed of 900 bp encoding 299 amino acid residues. Numbers on the right refer to nucleotides (top) and amino acids (bottom). The start (ATG) and stop (TAA) codons are underlined.

**Figure 3:**
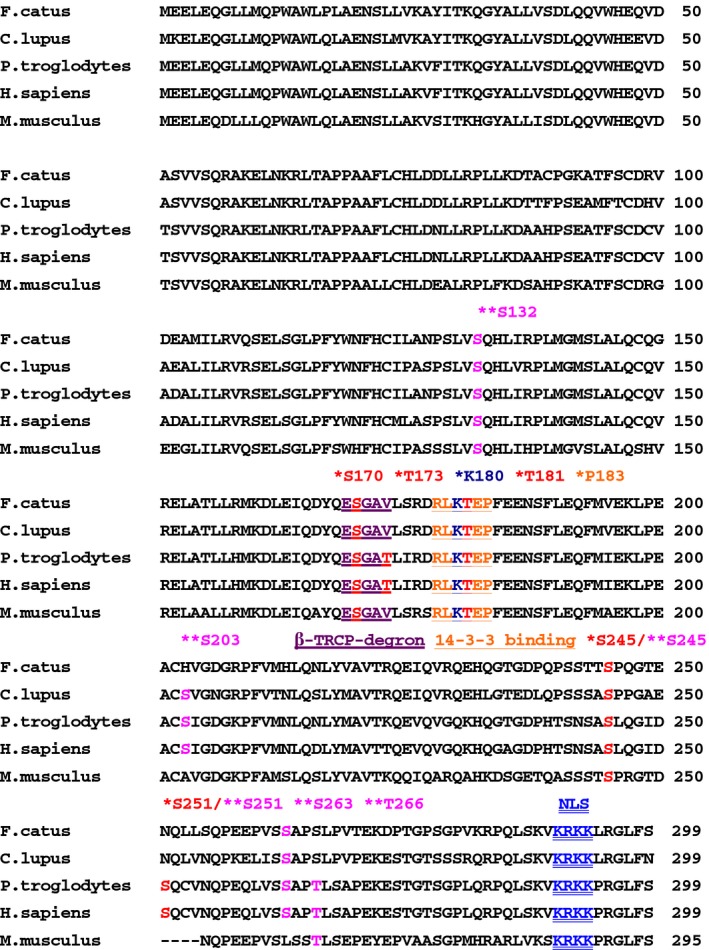
Sequence alignment of XLF. Amino acid sequences of XLF from cat (*Felis catus*, GenBank accession number: LC309246), dog (*Canis lupus familiaris*, GenBank accession number: LC176889), human (*Homo sapiens,* GenBank accession number: NM_024782.2), chimpanzee (*Pan troglodytes*, GenBank accession number: XM_001160321.5), and mouse (*Mus musculus*, GenBank accession number: NM_029342.4). The location of a putative β‐TRCP‐recognizable degron (β‐TRCP‐recognizable degron: _169_ ESGxT_173_), a putative 14‐3‐3 binding motif (14‐3‐3 consensus: _178_RxxT/SxP_183_), and a putative NLS sequence (NLS: _290_ KRKK_293_) in human XLF is shown 22, 24. The locations of the CKI phosphorylation sites (S170 and T173), AKT phosphorylation site (T181), putative 14‐3‐3 binding motif (P183), DNA‐PK phosphorylation site (S245), ATM phosphorylation site (S251) 24, 26, and ubiquitination site (K180) 28 in the human XLF sequence are marked with asterisks. The locations of the phosphorylation sites (S132, S203, S245, S251, S263, T266), which impact both the stability and DNA bridging capacity of XRCC4–XLF complexes in the human XLF sequence 25, are marked with a double asterisk.

The cDNA of the core NHEJ factors of feline species including *XLF* cDNA had not been cloned previously. Hence, we firstly cloned the feline *XLF* cDNA from a cat testis library and then sequenced it. We isolated a 900‐nucleotide open reading frame encoding the protein of 299 amino acids for the first time (Fig. 2). The feline *XLF* sequence has been deposited at the DDBJ/EMBL/NCBI database (accession number: LC309246). Comparative analysis of XLF sequences from different species showed that feline XLF had 80.9%, 84.9%, 82.3%, and 74.9% amino acid identity with the human, dog, chimpanzee, and mouse proteins, respectively. PTMs by phosphorylation, and protein–protein interactions of XLF as well as other core NHEJ factors might play a key role in the regulation of NHEJ pathways in human cells 21, 22, 23, 24, 25. As shown in Fig. 3, we found that the phosphorylation sites of DNA‐PK (S245), AKT‐1 (T181) and casein kinase I (CKI) (S170) in human XLF 24, 26 are evolutionarily conserved in cat, dog, chimpanzee, and mouse. On the contrary, the ataxia telangiectasia mutated (ATM) (S251) and CKI (T173) phosphorylation sites of human XLF 24, 26 are not conserved in cat, dog, and mouse. Most recently, Normanno *et al*. 25 reported that the simultaneous phosphorylation of six phosphorylation sites (S132, S203, S245, S251, S263, and S266) of human XLF and the eight phosphorylation sites of human XRCC4 might be important for the regulation of both the stability and DNA bridging capacity of XRCC4–XLF complexes. First, we found that the 266th amino acid of XLF is threonine in human, chimpanzee, and mouse, but serine in cat and dog, although the amino acid in human has been previously described as serine in 25. Regulatory mechanisms of subcellular localization of XLF in human and cow might play an important role in the control of the NHEJ activity 13, 22, 27. We described previously that the basic amino acids in the putative nuclear localization signal (NLS) of XLF are highly conserved among humans, chimpanzee, mouse, domestic animal species including cattle, goat, horse, birds, and dogs 13, 27. In this study, our data show that the putative NLS sequence is also conserved in cat (Fig. 3). It is reported that human XLF has a putative β‐transducin repeat containing protein (β‐TRCP)‐recognizable degron motif (_169_ESGAT_173_) and a putative 14‐3‐3 binding motif (_178_RLKTEP_183_) 24. We found that the 14‐3‐3 binding site (P183) of human XLF is evolutionarily conserved in cat, dog, chimpanzee, and mouse, whereas the putative β‐TRCP‐recognizable degron of human XLF is not conserved in cat as well as in dog and mouse XLF. Recently, it was reported that K180 in the putative 14‐3‐3 binding motif on XLF is a target for ubiquitination in human cells 28. We found that the target site is highly conserved among all five species examined including cat.

### Localization of XLF in feline cells

**Figure 4:**
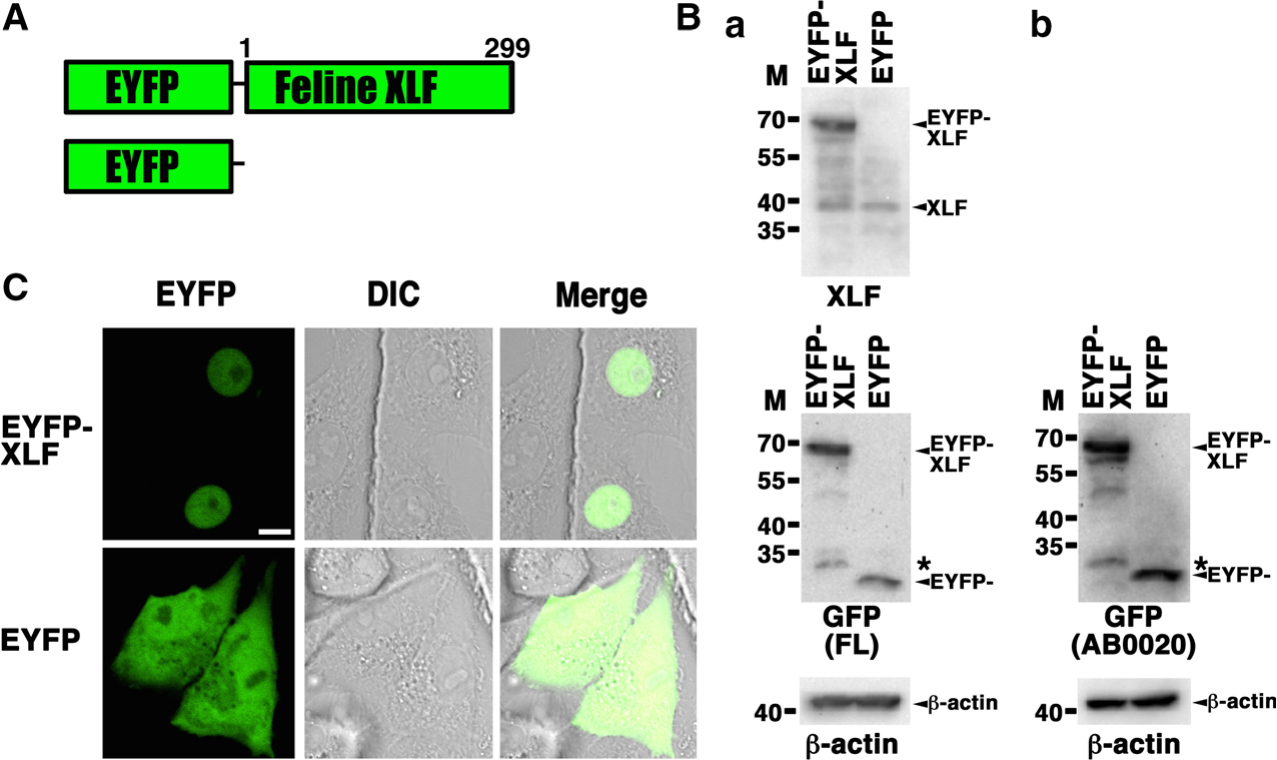
Localization of feline XLF. (A) Scheme showing the EYFP–feline XLF chimeric protein (EYFP‐feline XLF) and control protein (EYFP). (B) Expression of EYFP–feline XLF. Extracts from CRFK cells transiently expressing EYFP–feline XLF or EYFP were analyzed by western blotting using an anti‐XLF antibody (SAB2501119), two anti‐GFP antibodies (FL and AB0020), and an anti‐β‐actin antibody. Sequential detections by three antibodies (XLF, GFP and β‐actin) (Ba) or two antibodies (GFP and β‐actin) (Bb) were performed by reprobing on a blot. *The band might be a part of degradation products of GFP–feline XLF. M, molecular mass marker (kDa). (C) Imaging of live feline cells transfected with pEYFP‐feline *XLF*. CRFK cells transiently expressing EYFP–feline XLF or EYFP were examined by confocal laser microscopy. EYFP images for the same cells are shown alone (left panel) or merged (right panel) with the corresponding differential interference contrast (DIC) (center panel) images. Bar, 10 μm.

To investigate subcellular localization of XLF in live feline cells, we generated CRFK cells transiently expressing EYFP–feline XLF. For this purpose, the expression vector pEYFP‐C1 containing feline *XLF* (pEYFP‐feline *XLF*) was transfected into CRFK cells (Fig. 4A). Western blotting using an anti‐XLF and two anti‐GFP antibodies showed that the chimeric protein was expressed in the transfected feline cells (Fig. 4B). Confocal laser microscopy demonstrated that during interphase, EYFP–feline XLF localized in the nuclei of the cells transfected with pEYFP‐feline *XLF* (Fig. 4C). Expectedly, EYFP, used as a control, was distributed throughout the cell (with the exclusion of the nucleoli) in pEYFP‐transfected CRFK cells (Fig. 4C).

### Immediate accumulation of Ku‐dependent EYFP–feline XLF at laser‐microirradiated DSB sites

**Figure 5:**
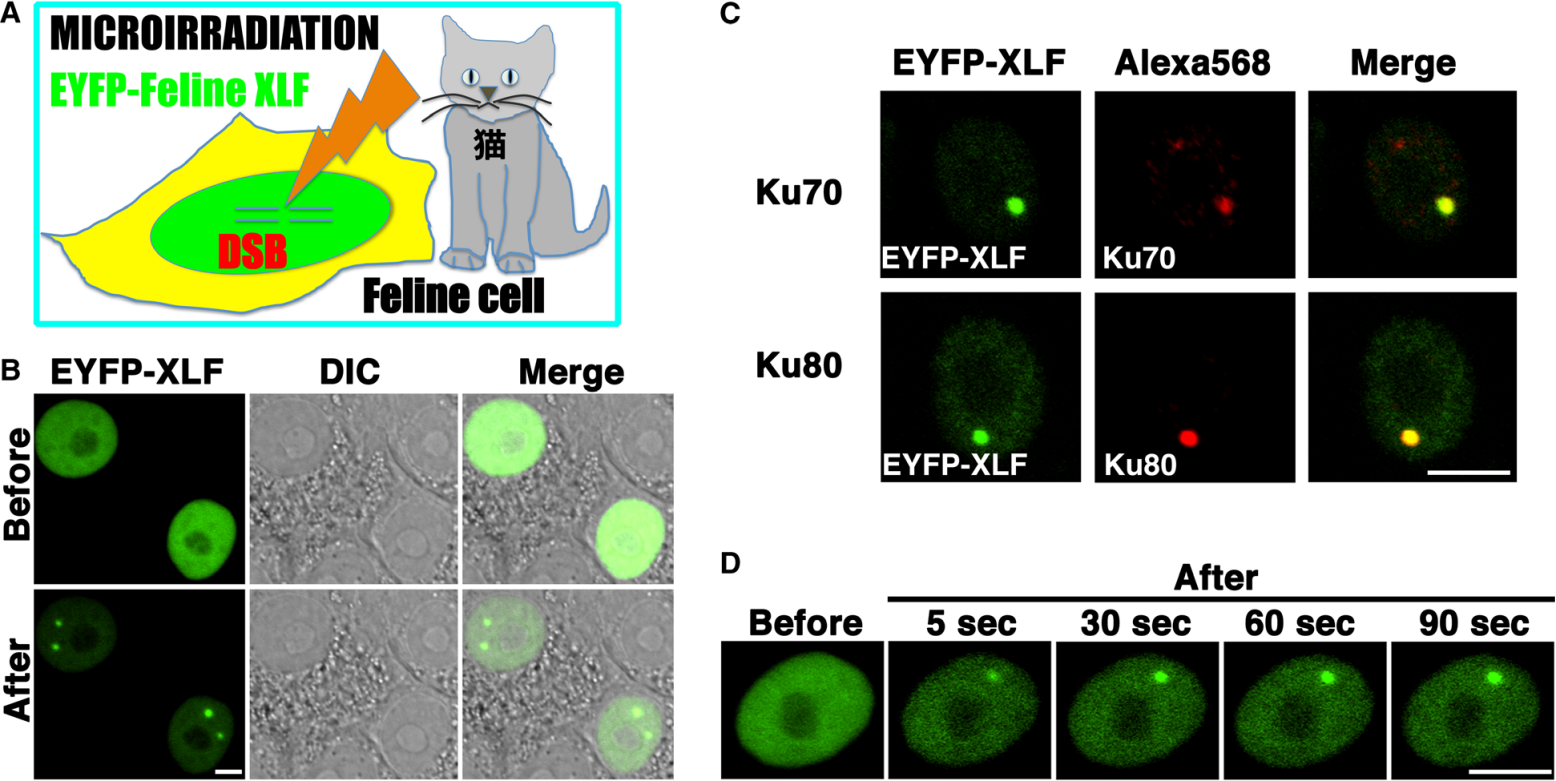
Accumulation of EYFP–feline XLF at the sites of DSBs induced by laser microirradiation. (A) The recruitment of EYFP–feline XLF to DSBs induced by 405 nm laser irradiation in CRFK cells. (B) Imaging of live CRFK cells transfected with pEYFP‐feline *XLF* before (upper panel) and after (bottom panel) microirradiation. EYFP images for the same cells are shown alone (left panel) or merged (right panel) with the corresponding DIC (center panel) images. (C) Immunostaining of microirradiated cells transfected with pEYFP‐feline *XLF* using an anti‐Ku70 antibody (H‐308) or anti‐Ku80 antibody (AHP317). Cells were fixed and stained with each antibody 5 min post‐irradiation. Left panel, EYFP–feline XLF; center panel, Ku70 (upper), Ku80 (bottom); right panel, merged images. (D) Time‐dependent EYFP–feline XLF accumulation in live CRFK cells, from 5 to 90 s after irradiation. Bar, 10 μm.

Previously, we and others have demonstrated that human core NHEJ proteins, such as Ku70, Ku80, XLF, and XRCC4, accumulate at DSB sites immediately after irradiation 19, 23, 29. It has never been investigated whether feline core NHEJ proteins accumulate at DSB sites immediately after DNA damage. We investigated whether, in feline cells, EYFP–feline XLF accumulates immediately at sites of DSB induced by laser microirradiation (Fig. 5A). Local DSBs in feline cells were induced using a 405 nm laser. Laser microirradiation resulted in the accumulation of EYFP–feline XLF at the microirradiated sites in live CRFK cells (Fig. 5B). To confirm if EYFP–feline XLF actually accumulated at the DSBs induced by the 405 nm laser, we immunostained the cells with an antibody against Ku70 or Ku80, a sensor of DSB. We found that EYFP–feline XLF accumulates and colocalizes with Ku70 and Ku80 at the DSB sites in CRFK cells (Fig. 5C). To examine the temporal dynamics of XLF localization, we carried out time‐lapse imaging in CRFK cells transfected with pEYFP‐feline *XLF*. We found that EYFP–feline XLF accumulates at the microirradiated sites 5 s after irradiation (Fig. 5D).

**Figure 6:**
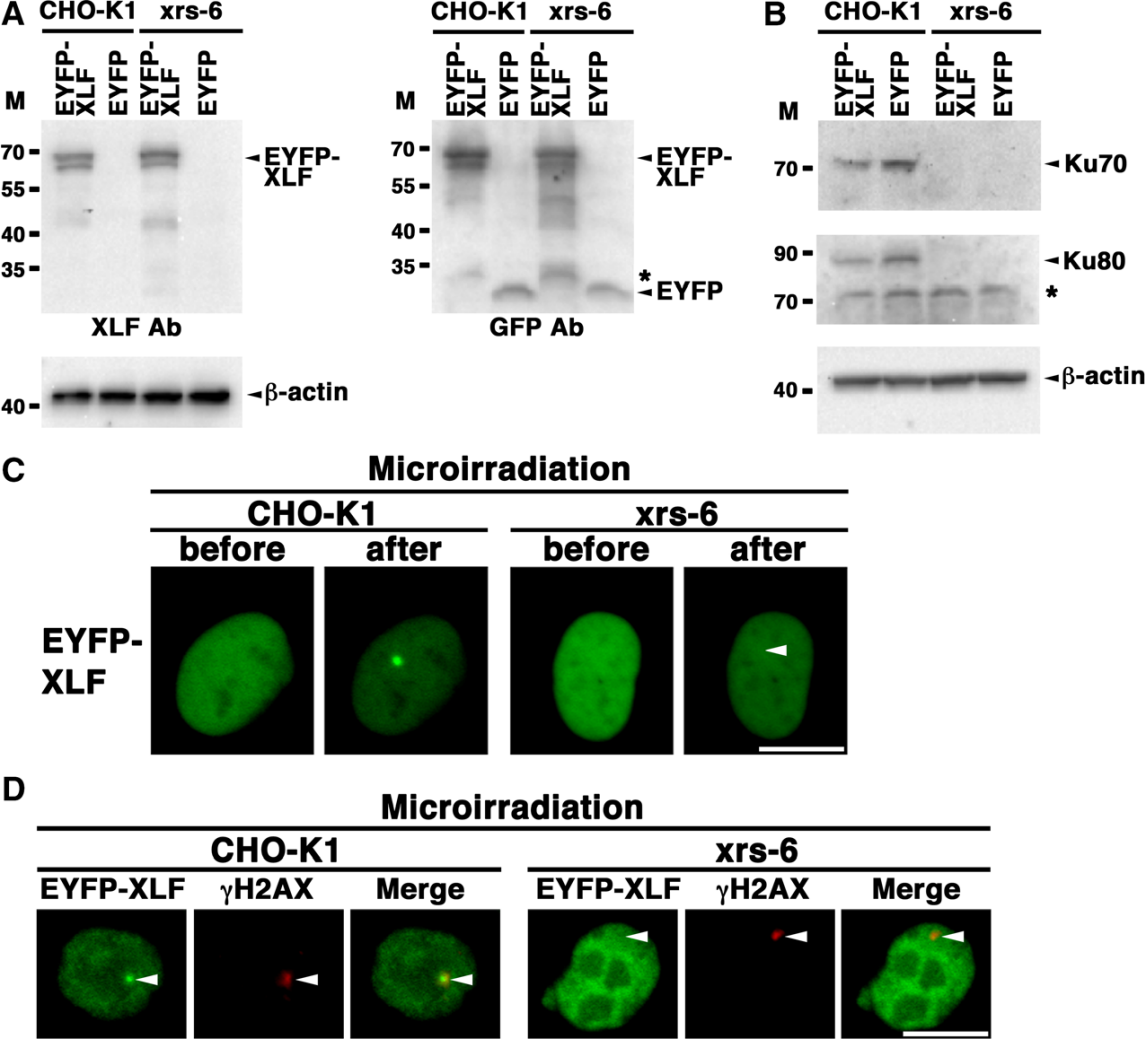
Ku‐dependent accumulation of EYFP–feline XLF at the sites of DSBs induced by laser microirradiation. (A,B) Expression of EYFP–feline XLF in the hamster ovary epithelial cell line (CHO‐K1) and the Ku80‐deficient CHO‐K1 mutant cell line (xrs‐6). Extracts (A, 7.5 μg/lane; B, 20 μg/lane) from each cell line transiently expressing EYFP–feline XLF or EYFP were analyzed by western blotting using anti‐XLF (SAB2501119) (A), anti‐GFP (FL) (A), anti‐Ku70 (H‐308) (B), anti‐Ku80 (AHP317) (B), and anti‐β‐actin antibodies (A,B). Sequential detections by three antibodies (XLF, GFP and β‐actin) were performed by reprobing on a blot (A). *The band might be a part of degradation products of EYFP–feline XLF (A). *Nonspecific band (B). M, molecular mass marker (kDa). (C) Feline XLF tagged with EYFP accumulated at irradiated sites in the CHO‐K1, but not in the Ku80‐deficient xrs‐6 cells. (D) Immunostaining of microirradiated cells transfected with pEYFP‐feline *XLF* using an anti‐γH2AX antibody. The CHO‐K1 or xrs‐6 cells were fixed and stained with each antibody 5 min post‐irradiation. Left panel, EYFP–feline XLF; center panel, γH2AX; right panel, merged images. Arrowheads indicate the microirradiated sites. Bar, 10 μm.

Next, we examined whether Ku (heterodimer of Ku70 and Ku80) is essential for the accumulation of feline XLF. We first confirmed that EYFP–feline XLF was expressed in both CHO‐K1 and Ku80‐deficient CHO‐K1 mutant cells (xrs‐6) transfected with pEYFP‐feline *XLF* (Fig. 6A,B). We found that EYFP–feline XLF does not accumulate at the microirradiated region in the xrs‐6 cells, whereas it accumulates in CHO‐K1 cells (Fig. 6C). We also confirmed whether EYFP–feline XLF accumulated at laser‐induced DSB sites by immunostaining the cells with an antibody that recognizes γH2AX. As shown in Fig. 6D, EYFP–feline XLF colocalized with γH2AX at the laser‐induced DSB sites in the CHO‐K1 cells, but not in the xrs‐6 cells, indicating that the recruitment of EYFP–feline XLF is dependent on the presence of hamster Ku in the hamster cells. Collectively, these results suggest that Ku is essential for the accumulation at DSBs of feline XLF.

## Discussion

In the treatment of cancers in humans and companion animals such as cats, resistance to chemotherapy and radiotherapy is a common and critical problem. The DSB repair proteins, especially core NHEJ factors, are charming target molecules for new anti‐cancer drugs 5, 6, 7, 8. Human XLF is one of the core NHEJ factors, and XLF‐deficient cells derived from human patients display ionizing radiation sensitivity 22, 23, 30. Hence, XLF is a possible target molecule for the development of next‐generation radiosensitizers and chemotherapeutics 5, 9. Chemo‐radiotherapy is the most common cancer treatment modality not only in human and dogs, but also in cats. Nevertheless, feline NHEJ and core NHEJ factors including XLF of cats remain to be investigated in depth. In this study, we cloned and sequenced the cDNA of feline *XLF*, and characterized feline XLF protein. Our data demonstrated that feline XLF localizes in the nuclei of feline CRFK cells, and feline XLF immediately accumulates at laser‐induced DSB sites, except in Ku‐deficient cells. Amino acid sequence alignment analysis for XLF suggested that the spatial and temporal control mechanisms of XLF, which play key roles in NHEJ, are evolutionarily conserved between humans and cats, although the structure of protein–protein interaction motifs and the sites of PTM are not perfectly conserved. These findings might be useful for clarifying the molecular mechanisms of NHEJ and of the radioresistance of feline cells 3, 4, and for the development of new radiosensitizers that target common targets against human and feline cancers.

In this study, our data demonstrated that feline XLF accumulates at DSB sites in a Ku‐dependent manner. Based on two impressive clinical successes of cancer treatment, DNA repair‐targeted therapies have attracted considerable attention. The first strategy utilizes the synthetic lethality targeting another remaining DNA repair pathway, e.g. by poly (ADP‐ribose) polymerase inhibitors for treating *BRCA1/2*‐mutated ovarian cancers 31. This strategy has provided a paradigm for many current clinical strategies targeting DNA repair. The second one is a new molecular targeted therapy employing immune checkpoint inhibitors including anti‐programmed cell death protein 1 (PD‐1) or anti‐programmed cell death 1 ligand 1 (PD‐L1) mAbs and resulted in dramatic clinical responses in certain types of solid malignancies, although the antitumor response rates were not enough 32. PD‐L1 is upregulated in the tumor cells after IR, and the adaptive response might mediate resistance to radiotherapy and treatment failure 33. On the basis of this, the authors proposed that irradiation and anti‐PD‐L1 treatment synergistically promote antitumor immunity 33. Recently, Sato *et al*. reported that Ku70/Ku80 depletion in cancer cells substantially enhances PD‐L1 upregulation after X‐irradiation 34, suggesting that the DSB repair pathway regulates PD‐L1 expression. These data suggest that further studies on the DSB repair mechanism in feline cells and the Ku‐dependent accumulation of feline XLF at DSBs will be useful for developing next‐generation drugs and combination therapies against cancers of both human and companion animals.

A substantial amount of data indicate that tumors with differential expression of the core NHEJ factors have differential therapy responsiveness 2, 35. To develop more effective DNA repair inhibitors and more efficient anti‐cancer drugs, it is important to clarify the molecular and control mechanisms of DNA repair pathways in feline cells. Unfortunately, however, no information is available about these in the context of feline cells. It is proposed that XLF proteins may be novel targets for human oral cancer stem cells, because their inhibition could lead to selective killing of these cells via spontaneous DSB induction and/or amplification of DNA damage following IR 9. Previously, our findings suggested that two predictive motifs, i.e. the NLS and 14‐3‐3 binding motif, needed for the regulation of human XLF subcellular localization are conserved among human, dog, mouse, and chimpanzee 13. In this study, our comparative analysis showed that feline XLF had only 80.9%, 84.9%, and 74.9% amino acid identity with the human, dog, and mouse protein sequences, respectively. We found that a ubiquitination target site (K180) for human XLF, which is on the putative 14‐3‐3 binding motif is perfectly conserved among cat, human, and the other three species. The ubiquitination target site might be important for the regulation of XLF localization and/or functions, although further studies are needed to confirm this. Collectively, these findings support the idea that the regulatory mechanism of subcellular localization of XLF is important for the control of XLF functions in human and mammalian cells, including those of cats.

Each core NHEJ factor is a potentially suitable molecular target for the development of chemotherapeutics and radiosensitizers for human cancers 2, 5, 6, 7, 8, 31, 35. Indeed, several core NHEJ factor‐targeted inhibitors including DNA‐PK inhibitors are in clinical development for human cancer treatment. However, as described above, there has been no report on the Ku‐dependent DNA repair mechanism via NHEJ in cats. Meanwhile, a number of studies showed that lack of XLF in mice can be compensated by Rag2, 53BP1, ATM, H2AX, DNA‐PKcs, Mri, and PAralog of XRCC4 and XLF (PAXX) 36, 37. Additionally, genetic interaction between XLF and PAXX was shown using human cells 38. In the present study, we firstly cloned and sequenced one of the core NHEJ genes, i.e. cDNA of feline *XLF*, and characterized feline XLF. Our data suggest that the mechanism of spatiotemporal regulation of XLF might be conserved in humans and cats. Our findings coupled with further studies might be useful to better understand the mechanism of the NHEJ and for the development of common DNA repair‐targeted drugs against cancers of both humans and cats.

## Conflict of interest

The authors declare no conflict of interest.

## Author contributions

MK and AK planned all experiments. MK, AK and YY performed experiments and interpreted data. MK wrote the manuscript, and AK and YY have read and approved the final manuscript.

## Data Availability

The sequence of feline XLF cloned in this study has been deposited to the DDBJ/EMBL/NCBI database [accession number: LC309246].
